# Autophagy-related gene 12 (ATG12) is a novel determinant of primary resistance to HER2-targeted therapies: Utility of transcriptome analysis of the autophagy interactome to guide breast cancer treatment

**DOI:** 10.18632/oncotarget.742

**Published:** 2012-11-17

**Authors:** Sílvia Cufí, Alejandro Vazquez-Martin, Cristina Oliveras-Ferraros, Bruna Corominas-Faja, Ander Urruticoechea, Begoña Martin-Castillo, Javier A. Menendez

**Affiliations:** ^1^ Metabolism and Cancer Group, Translational Research Laboratory, Catalan Institute of Oncology-Girona (ICO-Girona); ^2^ Molecular Oncology, Girona Biomedical Research Institute (IDIBGi); ^3^ Department of Medical Oncology, Breast Unit, Catalan Institute of Oncology (ICO)-Hospital Universitari de Bellvitge-Bellvitge Research Institute (IDIBELL); ^4^ Clinical Research Unit, Catalan Institute of Oncology-Girona (ICO-Girona)

**Keywords:** Autophagy, ATG genes, HER2, trastuzumab, lapatinib

## Abstract

The autophagic process, which can facilitate breast cancer resistance to endocrine, cytotoxic, and molecularly targeted agents, is mainly regulated at the post-translational level. Although recent studies have suggested a possible transcriptome regulation of the autophagic genes, little is known about either the analysis tools that can be applied or the functional importance of putative candidate genes emerging from autophagy-dedicated transcriptome studies. In this context, we evaluated whether the constitutive activation of the autophagy machinery, as revealed by a transcriptome analysis using an autophagy-focused polymerase chain reaction (PCR) array, might allow for the identification of novel autophagy-specific biomarkers for intrinsic (primary) resistance to HER2-targeted therapies. Quantitative real-time PCR (qRT-PCR)-based profiling of 84 genes involved in autophagy revealed that, when compared to trastuzumab-sensitive SKBR3 cells, the positive regulator of autophagic vesicle formation *ATG12* (autophagy-related gene 12) was the most differentially up-regulated gene in JIMT1 cells, a model of intrinsic cross-resistance to trastuzumab and other HER1/2-targeting drugs. An analysis of the transcriptional status of *ATG12* in > 50 breast cancer cell lines suggested that the *ATG12* transcript is commonly upregulated in trastuzumab-unresponsive HER2-overexpressing breast cancer cells. A lentiviral-delivered small hairpin RNA stable knockdown of the *ATG12* gene fully suppressed the refractoriness of JIMT1 cells to trastuzumab, erlotinib, gefitinib, and lapatinib in vitro. *ATG12* silencing significantly reduced JIMT1 tumor growth induced by subcutaneous injection in nude mice. Remarkably, the outgrowth of trastuzumab-unresponsive tumors was prevented completely when trastuzumab treatment was administered in an *ATG12*-silenced genetic background. We demonstrate for the first time the usefulness of low-density, autophagy-dedicated qRT-PCR-based platforms for monitoring primary resistance to HER2-targeted therapies by transcriptionally screening the autophagy interactome. The degree of predictive accuracy warrants further investigation in the clinical situation.

## INTRODUCTION

Although molecular drugs that specifically target one or several members of the HER signaling network can affect the expression and/or activation status of the HER2 oncoprotein, accumulating evidence suggests that the actual repercussions of HER2-targeting drugs (*e.g.,* the anti-HER2 monoclonal antibody trastuzumab or the dual HER1/HER2 tyrosine kinase inhibitor lapatinib) for tumor growth inhibition are closely related to the ability of these drugs to efficiently impede specific signaling pathways downstream of HER2 [1-8]. The identification of these pathways and whether they are operative before, during, and/or after treatment with HER2-inhibiting drugs might enable individual therapeutic decisions to be based on tumor biology rather than on basic histopathological data alone [9-23]. Autophagy (from the Greek *autos*, itself, and *phagein*, to eat) is a constitutive catabolic pathway that mediates both non-specific and targeted sequestration of cellular organelles and other macromolecules, which permits the degradation of cellular components in lysosomes and the recycling of bioenergetic metabolites [24-26]. The activation of autophagy might constitute a molecular mechanism that, beyond HER2 expression status in breast carcinoma cells, may accurately reflect the reliance of *HER2* gene-amplified breast carcinomas on HER2-driven signaling [27-29].

Previous studies have linked autophagy to both tumor-suppressive (*i.e.,* autophagy defects that predispose individuals to tumor development) and tumor-promoting functions (*i.e.,* autophagy activation promotes survival under stress, including cytotoxic chemotherapy) [30-35]. Of note, HER2 signaling and responsiveness to trastuzumab appear to dynamically interact with both the tumor-suppressive and tumorigenic roles of autophagy. The loss of *ATG6/Beclin1*, one of the earliest characterized mammalian autophagy genes that suggested a tumor suppression function for autophagy in certain mouse models of mammary oncogenesis [36-39], has been suggested to contribute to *HER2* gene amplification as well as to alterations in *PI3K* and *PTEN*, two crucial downstream effectors of HER2 [40]. Because the main predictors of trastuzumab activity in both pre-clinical and clinical settings are HER2 gene amplification and activation of the PI3K pathway, defined as *PTEN* loss and/or *PIK3CA* mutation [41-44], the loss of *ATG6/Beclin1* might function as a positive predictor of the tumor response to HER2-targeted therapies. Defective autophagy can therefore lead to enhanced cell death of HER2-positive cancer cells bearing competent apoptosis or senescence pathways upon challenge with HER2 inhibiting drugs [28]. However, we have added trastuzumab to the growing list of molecularly targeted therapeutics that activate the pro-survival function of autophagy as part of their mechanism of action [27]. Autophagic cell death, also referred to as active cell death II [APDC II], has been suggested to occur in response to extreme autophagic degradation triggered upon exposure to several cancer therapies [45-47], but it is not an obligatory outcome of trastuzumab-activated autophagy in a trastuzumab-responsive model of *HER2* gene-amplified breast carcinoma cells. Our studies additionally confirmed that the hyperactivation of basal autophagy was causally related to the acquisition of secondary resistance to trastuzumab [27]. Thus, “protective autophagy” causally enabled HER2 gene-amplified breast carcinoma cells to optimally grow for several months in the presence of clinically relevant concentrations of trastuzumab. The exploration of the cytoprotective effects of autophagy in breast cancer cells subjected to the pharmacologically mediated depletion of growth factor receptor signaling by mono-HER1 (*i.e.,* gefitinib, cetuximab), mono-HER2 (*i.e.,* trastuzumab), and dual HER1/HER2 (*i.e.,* lapatinib) inhibitors has confirmed that enhanced autophagic activity can contribute to more efficient maintenance of cancer cell resistance to HER targeting drugs [48-51]. However, a crucial aspect of this process that has remained unexplored is the possibility that *HER2* gene-amplified breast carcinoma cells might also exploit the cytoprotective role of autophagy to escape from HER2-targeted therapies *ab initio*.

It is well recognized that the autophagic process is mainly regulated at the post-translational level. However, a growing body of evidence has started to suggest that autophagy might also be regulated at the transcriptional level. We now have several methods and tools for the analysis of the autophagic flux and post-translational modifications of autophagy proteins [52]. Unfortunately, little is known about the analysis tools that can be applied to explore changes in the expression of autophagy genes or about the functional importance of putative candidate genes emerging from autophagy-dedicated transcriptome studies. In this context, we first assessed the utility of an autophagy-focused polymerase chain reaction (PCR) array to identify novel autophagy-specific gene biomarkers for intrinsic (primary) resistance to trastuzumab in *HER2* gene-amplified breast cancer cells that naturally exhibit *bona fide* primary resistance to HER-targeted therapies [12, 13, 17, 53-55]. Second, using molecular biology approaches we unambiguously validated whether the autophagy genes differentially expressed in trastuzumab-refractory breast carcinoma cells functionally predicted the primary response to the growth-inhibitory and anti-tumoral effects of trastuzumab. When employing pre-clinical models of trastuzumab-refractory HER2-overexpressing breast cancer cultures and xenografts, we were able to confirm that the transcriptional screening of the autophagy interactome can accurately identify autophagic pathway genes that operate as a primary mechanism of trastuzumab resistance in breast carcinoma cells.

## RESULTS

**Figure 1:**
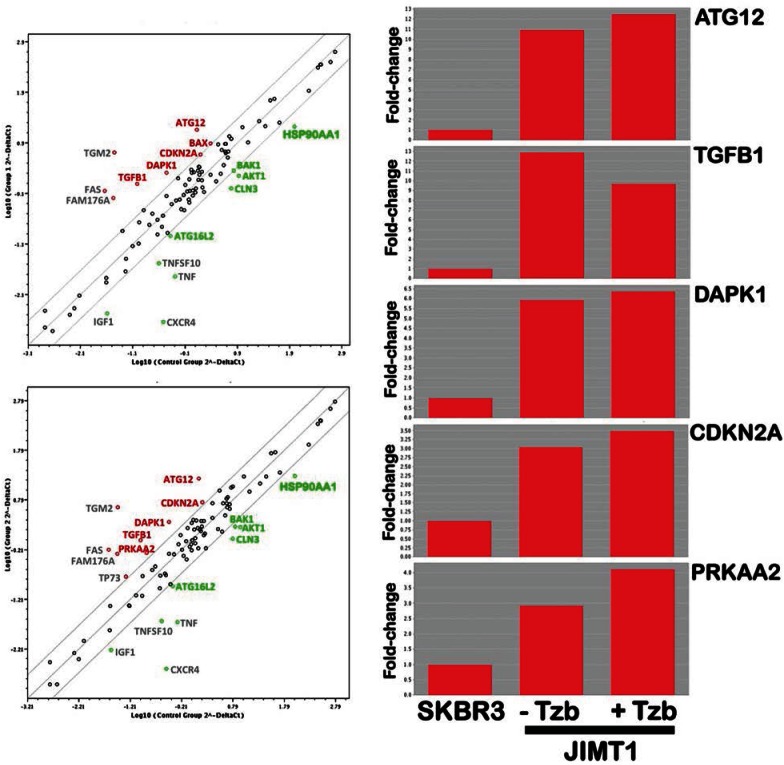
Analysis of autophagy genes in trastuzumab-refractory breast cancer cells *Left.* Total RNA from trastuzumab-sensitive SKBR3 and trastuzumab-refractory JIMT-1 cells was characterized in technical triplicates using the Autophagy RT^2^ Profiler PCR Array as per the manufacturer's instructions (SABiosciences; http://www.sabiosciences.com/rt_pcr_product/HTML/PAHS-084A.html). Representative scatter plots of the difference (≥ 2-fold; green and red symbols indicate downregulation and upregulation *vs.* expression levels in SKBR3 cells, respectively) in relative transcript abundance of 84 key genes involved in autophagy are shown. Grey symbols denote the fold-change results to be validated with a sufficient number of biological replicates [*i.e.,* fold-change results may have greater variations if the p-value > 0.05, or the p-value for the fold-change is either unavailable or relatively high (p > 0.05)] or that are uninterpretable because the gene's average threshold cycles were either not determined or were greater than the defined cut-off value (default 35) in both samples. *Total.* The transcript abundance of selected autophagy-related genes were calculated using the delta Ct method and presented as fold-change vs. basal expression in trastuzumab-sensitive SKBR3 cells.

Autophagy-focused PCR arrays indicate ATG12 as a candidate gene for primary (inherent) resistance to trastuzumab. We first explored whether there is a programmed series of genetic events that control the autophagic flux that could accompany *de novo* refractoriness to trastuzumab in *HER2* gene-amplified breast carcinoma cells. RNAs from trastuzumab-responsive SKBR3 cells, a widely employed *in vitro* tumor model characterized by naturally occurring *HER2* gene amplification, HER2 receptor protein overexpression, and HER2-dependency for cell proliferation and survival [18, 56, 57], and trastuzumab-refractory JIMT1 cells, a *HER2* gene-amplified cell line established from a ductal carcinoma pleural metastasis of a 62-year-old patient who did not respond to trastuzumab treatment *ab initio* [53-55], were evaluated by quantitative real-time PCR (qRT-PCR) to evaluate the expression of 84 key genes involved in autophagy (Fig. 1). When we imposed a two-fold change in mRNA expression level as the cut-off requirement to determine significant regulatory effects on autophagy-related genes, the autophagy suppressor *BAK1* (*BCL2-antagonist/killer 1*; ↓3-fold), the chaperone *HSP90AA1* (*Heat Shock Protein 90 kDa alpha [cytosolic], class A member 1*; ↓6-fold), the mTOR-related negative regulator of autophagy *AKT1* (↓6-fold), and the endosomal/lysosomal membrane protein-coding gene *CLN3* (*Ceroid-Lipofuscinosis, Neuronal 3*; ↓6-fold) were identified as down-regulated in trastuzumab-unresponsive JIMT1 cells compared to trastuzumab-responsive SKBR3 cells. The metabolic master rheostat protein *PRKAA2* (*AMP-activated Protein Kinase catalytic subunit alpha-*2; ↑3-fold), the retinoblastoma-related senescence/autophagy marker *CDKN2A/p16* (*cyclin-dependent kinase inhibitor 2*; ↑3-fold), the autophagy stimulator *DAPK1* (*Death-Associated Protein Kinase 1*; ↑6-fold), the multifunctional cytokine *TGFβ1* (Transforming Growth Factor-β1; 13-fold), and the essential autophagy gene *ATG12* (Autophagy-related 12 homolog (*S. cerevisiae*); ↑11-fold) were significantly up-regulated in trastuzumab-refractory JIMT1 cells. Trastuzumab exposure, while incapable of influencing the proliferation of JIMT1 cells, slightly enhanced the differential overexpression of the autophagy-related genes *PRKAA2*, *CDKN2A/p16*, *DAPK1* and *ATG12* (Fig. 1).

**Figure 2:**
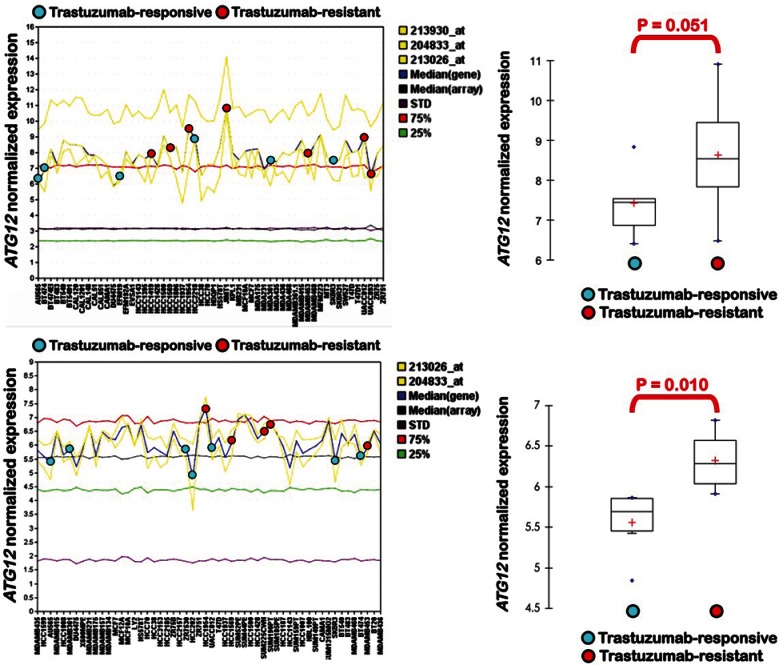
Differential expression of ATG12 in trastuzumab-responsive and trastuzumab-resistant breast cancer cell lines Distribution of *ATG12* gene expression in trastuzumab-responsive and trastuzumab-resistant breast cancer cell lines across the Adai data set (*top*) and the Neve's data set (*bottom*).

ATG12 is differentially up-regulated in HER2 gene-amplified breast carcinoma cells that exhibit primary (inherent) resistance to trastuzumab. To exclude JIMT1 cell line-specific effects and to further confirm that the differential overexpression of the *ATG12* gene might correlate with breast cancer cell phenotypes of primary (inherent) resistance to trastuzumab, we took advantage of the ROCK online interface, a publicly accessible portal that enables the rapid integration of breast cancer functional and molecular profiling datasets [58]. We analyzed the transcriptional profile of the *ATG12* gene across the Adai (GSE1090) gene expression dataset, which includes 56 breast cancer cell lines, and across the Neve's gene expression data set, which includes 54 widely used breast cancer cell lines [59]. The HER2-positive breast cancer cell lines were classified as trastuzumab-sensitive or trastuzumab-refractory based on the data from the literature. We then compared the normalized expression data for *ATG12* among trastuzumab-sensitive (*blue dots* in Fig. 2) and trastuzumab-refractory (*red dots* in Fig. 2) cell lines. When the expression status of *ATG12* was interrogated in the Adai data set, an increasing trend in mean *ATG12* mRNA expression values was observed in the trastuzumab-refractory group, which proved to be almost statistically significant (P = 0.051; Fig. 2). This trend reached statistical significance when analyzing the differences in *ATG12* gene expression between the trastuzumab-refractory and the trastuzumab-sensitive groups in the Neve's data set (P=0.010; Fig. 2).

Small hairpin RNA-driven stable knockdown of ATG12 sensitizes JIMT1 cells to trastuzumab and eliminates cross-resistance to HER1/HER2 tyrosine kinase inhibitors in vitro. To molecularly establish a causal role of ATG12-driven autophagy in the *de novo* resistance of *HER2* gene-amplified breast cancer cells to trastuzumab, we employed a lentivirus-mediated stable knockdown of the *ATG12* gene. To control for any off-target effects of short hairpin (sh)RNA transduction, control (negative) lentiviral particles were also used strictly in parallel. First, we determined if the expression of ATG12 was significantly *silenced* in JIMT1 cells that were stably transduced with ATG12-shRNAs. Lysates were produced from JIMT1 cell cultures stably expressing shRNAs that were designed to recognize and reduce the expression of ATG12 as well as from JIMT1 cells stably transduced with an empty vector (control shRNA). Immunoblotting analyses confirmed that ATG12 protein expression was reduced to almost undetectable levels (>95% reduction) in JIMT1 cells that were lentivirally transduced with ATG12 shRNAs compared to parental cells transduced with empty vector (see Ref. 60). All subsequent experiments were performed with these stable cell lines, which were termed control-shRNA/JIMT1 and ATG12-shRNA/JIMT1.

**Figure 3:**
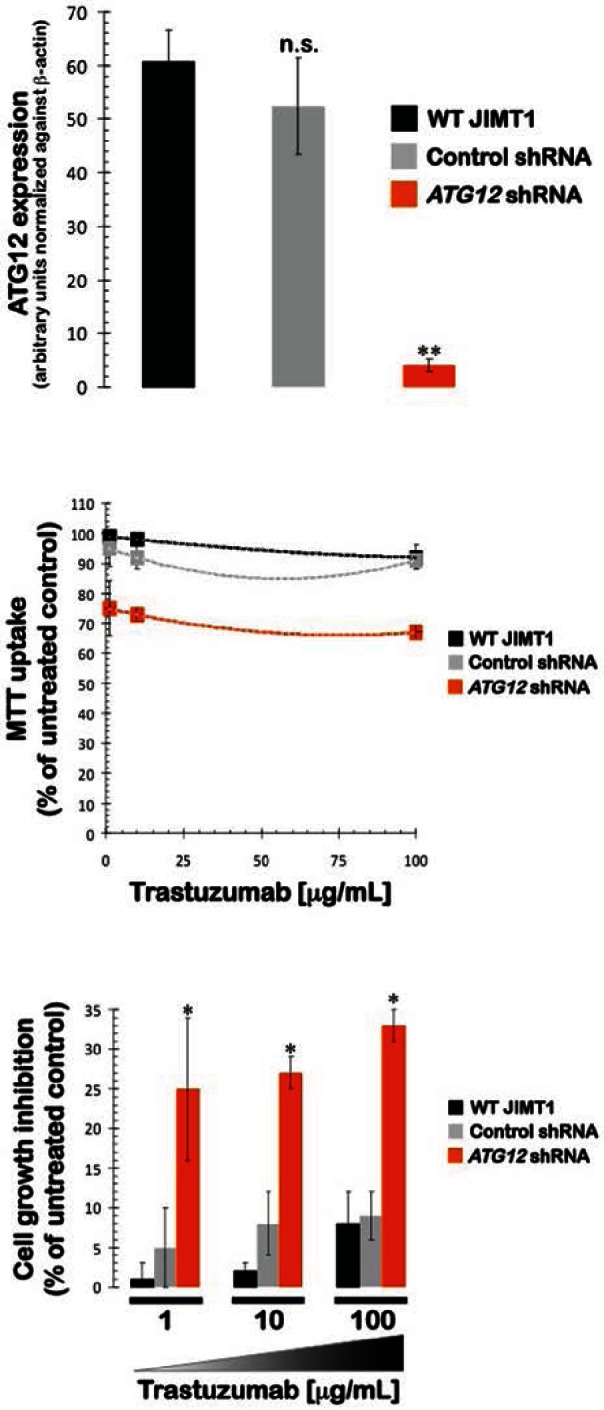
Impact of shRNA-driven genetic ablation of ATG12 on the efficacy of the anti-HER2 monoclonal antibody trastuzumab (Herceptin) in vitro The metabolic status of JIMT1 parental cells, control shRNA-JIMT1 cells, and ATG12 shRNA-JIMT1 cells treated with graded concentrations trastuzumab was evaluated using MTT-based cell viability assays, followed by the generation of dose-response graphs depicting the % of untreated cells (untreated control cells = 100% cell viability). The results are presented as the means (*columns*) and 95% confidence intervals (*bars*) of three independent experiments performed in triplicate. [n.s. not significant; * P < 0.05]

MTT-based cell viability assays were performed to evaluate the ability to reduce cell growth in JIMT1 cells before and after the stable knockdown of *ATG12*. Non-transduced JIMT1 parental cells, control-shRNA/JIMT1 cells, and ATG12-shRNA/JIMT1 cells were treated with various concentrations of trastuzumab for five days. We failed to obtain half-maximal inhibitory concentrations (IC_50_) in both the parental and control-shRNA/JIMT1 cells, thus confirming the robust resistance of JIMT1 cells to trastuzumab-mediated cytotoxicity (Fig. 3). ATG12-shRNA/JIMT1 cells, however, demonstrated a significantly enhanced sensitivity to trastuzumab at concentrations as low as 1 μg/mL. Thus, the insignificant levels of cell viability loss induced by trastuzumab in both the parental and control-shRNA/JIMT1 cells (*i.e.,* 1% reduction at 1 μg/mL trastuzumab and 8% reduction at 100 μg/mL trastuzumab, respectively) were augmented by up to 25-fold (*i.e.,* from 25% reduction at 1 μg/mL trastuzumab to 33% reduction at 100 μg/mL trastuzumab, respectively) in ATG12-shRNA/JIMT1 cells (Fig. 3).

**Figure 4:**
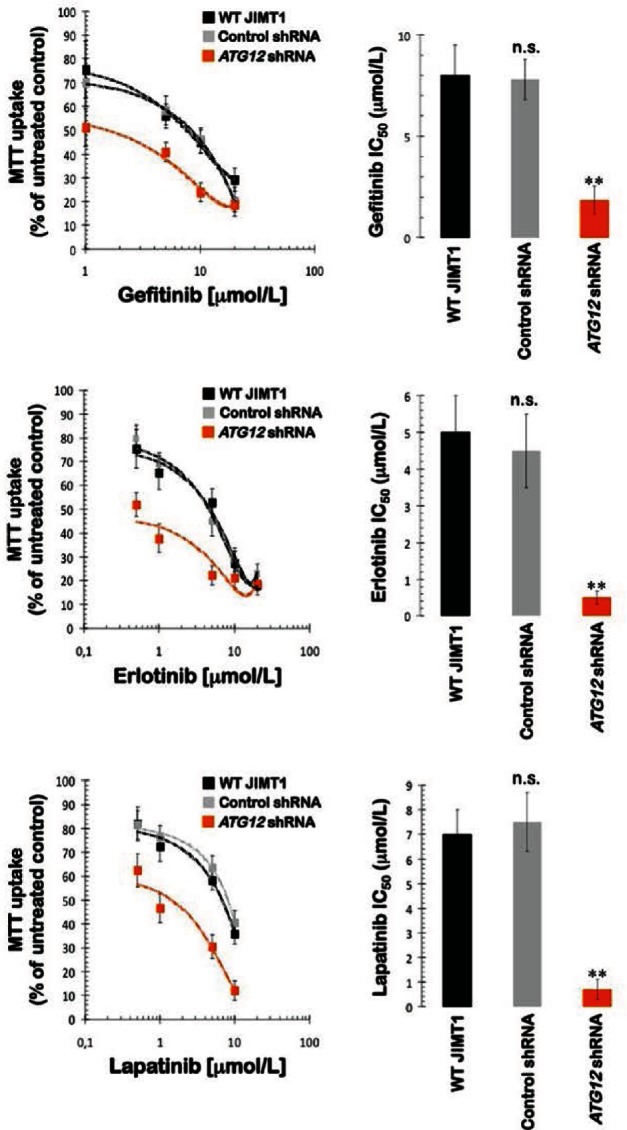
Impact of shRNA-driven genetic ablation of ATG12 on the efficacy of HER1/2-tyrosine kinase inhibitors in vitro *Left panels.* The metabolic status of JIMT1 parental cells, control shRNA-JIMT1 cells, and *ATG12* shRNA-JIMT1 cells treated with graded concentrations of erlotinib, gefitinib, and lapatinib was evaluated using MTT-based cell viability assays, followed by the generation of dose-response graphs depicting the % of untreated cells (untreated control cells = 100% cell viability). The results are presented as the means (*columns*) and 95% confidence intervals (*bars*) of three independent experiments performed in triplicate. *Right panels.* The degree of sensitivity of JIMT1 parental cells, control shRNA-JIMT1 cells, and *ATG12* shRNA-JIMT1 cells to HER1/2-targeting drugs is illustrated by bars representing mean IC_50_ values in each cell line. Error bars show standard deviations. [n.s. not significant; ** P < 0.005]

Because we recently demonstrated that the IC_50_ values for the small molecule HER1/HER2 TKIs gefitinib, erlotinib, and lapatinib were drastically higher (up to 40-fold) in trastuzumab-refractory JIMT1 cells than in trastuzumab-sensitive SKBR3 cells [17], we sought to evaluate whether the specific knockdown of ATG12 was sufficient to eliminate the inherent cross-resistance of JIMT1 cells to HER targeting drugs. Remarkably, the IC_50_ values for gefitinib, erlotinib, and lapatinib were drastically reduced as a result of *ATG12* gene silencing (Fig. 4). The gefitinib IC_50_ value decreased from 8.0±1.5 μmol/L and 7.8±1 μmol/L in parental and control-shRNA/JIMT1 cells, respectively, to 1.85±0.7 μmol/L in ATG12-shRNA/JIMT1 cells (*i.e.,* ≈4-fold decrease). The erlotinib IC_50_ value decreased from 5.0±1.0 μmol/L and 4.5±1.0 μmol/L in parental and control-shRNA/JIMT1 cells, respectively, to 0.5±0.2 μmol/L in ATG12-shRNA/JIMT1 cells (*i.e.,* ≈10-fold decrease). The lapatinib IC_50_ value decreased from 7.0±1.0 μmol/L and 7.5±1.0 μmol/L in parental and control-shRNA/JIMT1 cells, respectively, to 0.7±0.4 μmol/L in ATG12-shRNA/JIMT1 cells (*i.e.,* ≈10-fold decrease). Of note, the sensitizing effect of *ATG12* knockdown was restricted to HER targeting drugs because the growth inhibitory responses of ATG12-shRNA/JIMT1 cells to various cytotoxic molecules (*i.e.,* doxorubicin, 5-fluorouracil, cisplatin, vinorelbine, and paclitaxel) remained unaltered (data not shown).

**Figure 5:**
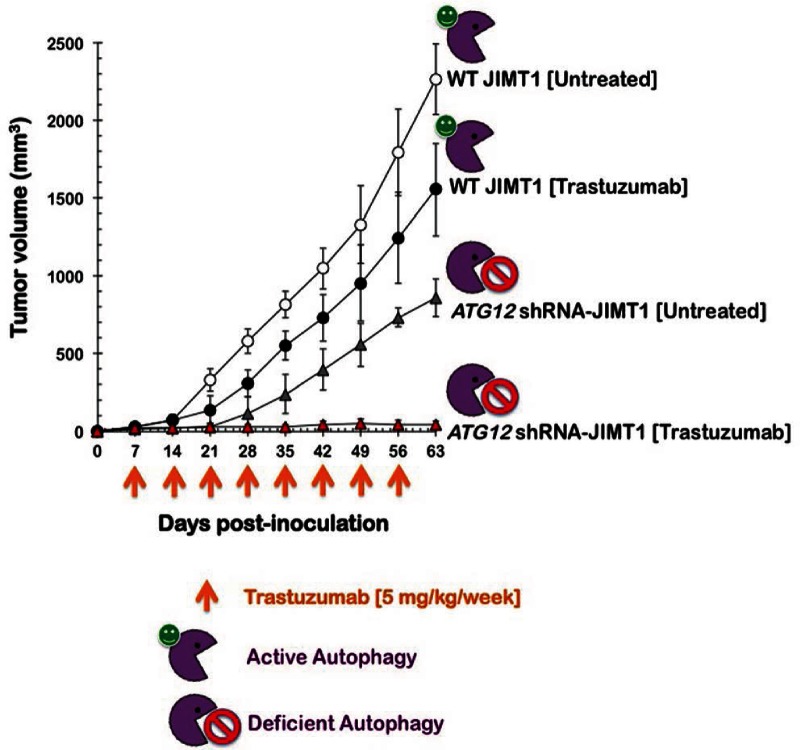
Impact of shRNA-driven genetic ablation of *ATG12* on the efficacy of trastuzumab *in vivo* Shown are the mean tumor volumes (±SD) in JIMT1 and ATG12 shRNA-JIMT1 xenograft-bearing nude mice following injection with trastuzumab (5 mg/kg/week) for nine weeks. Data from control shRNA-JIMT1 cells were superimposable with those obtained in JIMT1 parental cells and have been omitted for simplicity.

ATG12 knockdown reduces tumor growth and sensitizes trastuzumab-resistant xenografts to trastuzumab. The effects of the specific *ATG12* gene knockdown on tumor growth were investigated *in vivo* in a JIMT1 xenograft animal model (Fig. 4). Compared to the untreated control group (2266±228 mm^3^), nine weeks of treatment with trastuzumab (5 mg/kg/week) failed to prevent tumor growth by the parental JIMT1 cells; the mean tumor size reached volumes as large as 1556±298 mm^3^ in the presence of trastuzumab. However, compared to the mean xenograft tumor size in both the shRNA-control and trastuzumab-treated groups, the mean tumor size of the ATG12-shRNA/JIMT1 cells was smaller (858±121 mm^3^). Remarkably, when the ATG12-shRNA/JIMT1 xenografts were treated with trastuzumab weekly, the mean tumor size was drastically reduced to 46±21 mm^3^, thus demonstrating that the sensitivity of trastuzumab-refractory JIMT1 xenografts to trastuzumab increased dramatically when the cells were depleted of the autophagy driver ATG12. Whereas trastuzumab treatment reduced the tumor volume of ATG12-expressing JIMT1 xenografts 63 days post-injection by approximately 30%, trastuzumab exposure reduced the tumor volume of ATG12-silenced JIMT1 xenografts by an impressive 98% over the same treatment time (Fig. 5).

## DISCUSSION

We have previously reported that HER2-overexpressing breast cancer cells that have been chronically exposed to the anti-HER2 monoclonal antibody trastuzumab exhibit a *bona fide* up-regulation of the basal autophagic activity that efficiently operates to protect breast cancer cells from the growth-inhibitory effects of trastuzumab [27]. Our working model proposed that enhanced autophagosome formation and the subsequent catabolic function of autophagy flux actively contribute to the survival of HER2-dependent breast cancer and facilitate the rapid development of resistance to trastuzumab in cells that initially respond to treatment. Consequently, the blockade of the autophagic machinery should significantly help to prevent or retard the appearance of acquired (secondary) resistance to trastuzumab in *HER2* gene-amplified breast carcinoma cells [27, 28]. However, whether HER2-positive breast cancer cells might also exploit the cytoprotective role of autophagy to escape from HER2-targeted therapies *ab initio* was unexplored. Taking advantage of the *HER2* gene-amplified JIMT1 cell line [17, 53-55], which inherently exhibits cross-resistance to multiple HER1/2 inhibiting drugs, we have provided evidence for the first time that one of the ubiquitin-like conjugation systems that is requisite for the formation of autophagic vesicles (*i.e.,* ATG12) [61-66] is a novel determinant of primary resistance to HER1/2-targeted therapeutics.

In this study, we first analyzed the correlation between primary resistance to trastuzumab and the expression status of several genes that regulate distinct molecular events leading to autophagic vesicle formation [61-63, 67-70], including its initiation (*e.g.,* beclin1) and maturation by the ATG12 (*e.g.,* ATG12 and ATG5) and LC3 (*e.g.,* ATG4B) conjugation systems. We demonstrated that *DAPK1*, *TGFB1*, and *ATG12* are the three pro-autophagic genes with the highest differential up-regulation (>5-fold) between trastuzumab-unresponsive JIMT1 cells and trastuzumab-sensitive SKBR3 cells. The death-associated protein kinase-1 (DAPK1) induces autophagy and apoptosis through independent mechanisms [71, 72]. Autophagy induced by DAPK-1 overexpression can be attenuated by knocking down expression of the microtubule-associated protein MAP1B [72], suggesting that the interaction of DAPK-1 with MAP1B, which in turn is capable of interacting with LC3, might be responsible for the DAPK-1-driven induction of autophagy. The extremely pleiotropic behavior of DAPK-1 [73] also suggests that transcriptional programs influenced by DAPK-1 might also impact the propensity of cells to undergo autophagy. TGFβ, the best-characterized inducer of the epithelial-to-mesenchymal transition (EMT), has been shown to induce the accumulation of autophagosomes and the autophagic conversion of LC3 while enhancing the degradation of long-lived proteins [74, 75]. TGFβ-induced autophagy appears to contribute to the growth inhibitory and tumor suppressive effects of TGFβ, in conjunction with other anti-proliferative pathways downstream of TGFβ signaling. Of note, DAPK-1 is commonly silenced in human cancers by methylation [73] and has tumor and metastasis suppressor properties. DAPK-1- and TGFβ-driven autophagy, therefore, can be viewed as dual-model paradigms in which autophagy may contribute to tumor suppression early in the development of a subset of HER2 gene-amplified breast carcinomas; however, when these tumors somehow bypass the tumor suppressor activity of DAPK-1 and TGFβ, autophagy would later allow the selection of invasive, mesenchymal cells with an enhanced expression of autophagic and stem cell markers. In breast cancer, the undifferentiated CD44^+^CD24^−/low^ antigenic state commonly attributed to breast cancer subpopulations with cancer stem cell (CSC) properties is highly enriched with EMT transcriptional factors and mesenchymal markers [76-82]. Of note, our group has recently confirmed that a) CD44^+^CD24^−/low^ mesenchymal subpopulations are intrinsically unresponsive to trastuzumab [83]; b) the absence or presence of the CD44^+^CD24^−/low^ mesenchymal immunophenotype is closely related to the inherent sensitivity or refractoriness of HER2-gene amplified breast carcinoma cells to trastuzumab [83], respectively; and c) the impairment of the autophagic flux by genetic means (i*.e., ATG12* gene silencing) drastically decreases the number of trastuzumab-unresponsive cells bearing CD44^+^CD24^−/low^ cell surface antigens and appears to impede the ontogeny of generating the CSC-like CD44^+^CD24^−/low^ mesenchymal phenotype by preventing the full acquisition of a post-EMT status [60]. These findings, together with our current description that a) ATG12 overexpression is a new molecular biomarker for primary resistance to trastuzumab and b) inherent unresponsiveness to trastuzumab can be switched to sensitivity simply by depleting ATG12 notably delineates a new scenario in which primary resistance to HER2-targeted drugs can be understood in terms of *HER2* gene-amplified breast carcinoma cells that are intrinsically capable of generating highly autophagic, undifferentiated CD44^+^CD24^−/low^ antigenic states. Importantly, when investigating the relationship between the biological roles of autophagy in trastuzumab-mediated cancer therapy, we concluded that ATG12-related autophagic flux specifically functions as a protective cellular phenotype against HER-targeting drugs. Whereas the IC_50_ values for the small-molecule HER1/2 TKIs erlotinib, gefitinib, and lapatinib were drastically reduced by up to 10-fold in response to *ATG12* gene silencing, the knockdown of *ATG12* failed to alter the sensitivity of JIMT1 cells to multiple cytotoxics, including doxorubicin, 5-fluorouracil, cisplatin, vinorelbine, and paclitaxel (data not shown). Autophagy addiction underlying the primary resistance of HER2 gene-amplified breast cancer cells to HER1/2 targeting therapies may depend, at least in part, on the previously unrecognized ability of autophagy to regulate a trastuzumab-unresponsive CD44^+^CD24^−/low^ mesenchymal cellular state.

Autophagy-addicted, trastuzumab-unresponsive JIMT1 cells significantly upregulated the expression of *PRKAA2*, the catalytic subunit of the master regulator of cellular energy homeostasis AMPK [84-86]. Cancer cells are largely unable to change their metabolic programming as a result of their oncogenic alterations [87-91], which may underlie the sensitivity of HER2-overexpressing breast cancer cells to growth factor receptor inhibitors, including trastuzumab and lapatinib. Indeed, the lapatinib-induced activation of AMPK and the consequent inhibitory effects on protein synthesis and *de novo* fatty acid biogenesis are lethal to *HER2* gene-amplified breast carcinoma cells that are “addicted” to glycolysis [92, 93]. Lapatinib treatment seems to deprive cells of energy not only *via* the down-regulation of glycolysis but also by further triggering an entire cascade of metabolic events mediated by the activation of AMPK. AMPK activation ultimately results in downstream events that conserve cellular energy by inhibiting fatty acid synthesis while activating fatty acid oxidation as an alternative source of energy. Intriguingly, the ability of AMPK to coordinate the adaptive response of cellular bioenergetics to ATP depletion may also be responsible, at least in part, for the reduced sensitivity of *HER2* gene-amplified breast cancer cells to HER-targeting drugs. Given that AMPK modulates the activity of crucial regulators of autophagy such as mTOR, increased expression of AMPK accompanied by the downregulation of key negative regulators of mTOR (*i.e.,* AKT1) may aid trastuzumab-refractory breast cancer cells in mounting a protective autophagic response from a molecular perspective. The chronic elevation of AMPK and mTOR activities likely results in a shift toward catabolism, which can inherently *prepare* HER2-overexpressing cells to accommodate the acute bioenergetic crisis imposed by HER-targeting drugs. This AMPK-related metabolic scenario is likely associated with the occurrence of autophagic, trastuzumab-refractory CD44^+^CD24^−/low^ mesenchymal immunophenotypes because treatment with metformin, a partial inhibitor of autophagy due its limited ability to strongly reduce intracellular ATP [94-96], is sufficient to efficiently kill EMT-related CD44^+^CD24^−/low^ cells and restore trastuzumab efficacy in *HER2* gene-amplified breast cancer cells with primary resistance to HER1/2-targeting therapies [18, 97-101].

*A priori* autophagic flux is a crucial feature that determines the efficacy of HER2-directed drugs *ab initio*. Targeting autophagy addiction may be helpful in circumventing the primary resistance to HER2 targeting therapies and may potentiate the efficacy of HER2 targeting strategies currently in use in HER2-positive breast cancer patients. While ATG12 is not currently a druggable target, emerging autophagy inhibitors (*e.g.,* Lys05, Spautin-1) that target other components of the autophagic vesicle assembly [102-104] may prove to be an effective means to circumvent primary resistance to and potentiate the efficacy of HER2-targeting strategies currently in use in HER2-positive breast cancer patients. All human clinical trials currently exploring autophagy inhibition as a therapeutic strategy employ chloroquine or its derivative hydroxychloroquine due to the long track record of safety in human patients [105-107]. However, recent studies suggest that hydroxychloroquine is not a potent autophagy inhibitor at clinically tolerable doses and that chloroquine-mediated lysosomal dysfunction sensitizes breast cancer cells to chemotherapy independent of autophagy [108]. Despite this unfavorable scenario, we are currently exploring whether chloroquine-induced inhibition of the final step in the autophagic pathway sensitizes autophagy-addicted [109], trastuzumab-refractory HER2-positive breast cancer cells to the growth inhibitory activity of trastuzumab. Preliminary evidence appears to support a valuable pre-clinical scenario in which lysosomotropic autophagy inhibitors (*e.g.,* chloroquine and its derivatives) [105] could prove to be more effective and autophagy-specific when combined with molecularly targeted drugs (*e.g.,* trastuzumab) whose mechanisms of action involve HER2 internalization and recycling [110-113] *via* chloroquine-targeted lysosomal pathway-dependent degradation.

Using very simple bioinformatics tools on well-established cell-based breast carcinoma models, we demonstrated for the first time the usefulness of low-density, autophagy-dedicated qRT-PCR-based platforms for monitoring primary trastuzumab resistance by transcriptionally screening the autophagy interactome. The autophagy qRT-PCR tool used on trastuzumab-refractory breast cancer cells allowed for the rapid and accurate identification of differentially expressed autophagy genes (*e.g., ATG12*) that were causally associated with intrinsic resistance to the anti-tumoral effects of the molecularly targeted anti-HER2 drugs. While our current ability to accurately measure basal autophagic flux or determine whether autophagy is activated or inhibited in patient samples (*i.e.,* immunohistochemical- or immunoblotting-based detection of autophagic markers, such as LC3 and SQSTM1/p62) is very limited and of unproven utility in human patients, the method of analysis described here or other similar technologies involving human autophagy-dedicated cDNA microarrays [114], by allowing the simultaneous monitoring of the expression level of multiple autophagy genes, might provide a practical method for analyzing the impact of the autophagy interactome in the *in vivo* response of breast cancer tissues to anti-cancer therapies. The degree of accuracy that constitutive activation of autophagy, as revealed by a transcriptome analysis using PCR arrays, can provide to predict trastuzumab efficacy warrants further investigation in the clinical situation.

## METHODS

### Drugs

The EGFR (HER1)-Tyrosine Kinase Inhibitor (TKI) gefitinib (ZD1839; Iressa^®^) was kindly provided by AstraZeneca (AstraZeneca PLC Headquarters, 15 Stanhope Gate, W1K 1LN, London, UK). The EGFR (HER1) TKI erlotinib (Tarceva^®^) was a kind gift from Roche Pharmaceuticals (Neuilly sur Seine, France). The dual HER1/HER2-TKI1 lapatinib (GW572016; Tykerb^®^) was kindly provided by GlaxoSmithKline (GSK), Corporate Environment, Health & Safety (Brentford, Middlesex TW8 9GS, UK). Stock solutions of gefitinib, erlotinib, and lapatinib (10 mmol/L) were prepared in DMSO and stored in aliquots in the dark at −20°C until use. Trastuzumab (Herceptin^®^) was kindly provided by Hospital Universitari de Girona Dr. Josep Trueta Pharmacy (Girona, Spain). Trastuzumab was solubilized in bacteriostatic water for injection containing 1.1% benzyl alcohol (stock solution at 21 mg/mL), stored at 4°C and used within 1 month. For experimental use, all experimental agents were prepared fresh from stock solutions and were diluted with cell growth medium. Control cells were cultured in media containing identical concentrations (v/v) as that used for test cells. The vehicle solutions had no noticeable influence on the proliferation of experimental cells.

### Cell lines and culture conditions

JIMT1 cells were obtained from the German Collection of Microorganisms and were routinely grown in Dulbecco's modified Eagle's medium (DMEM, Gibco^®^ Cell Culture Systems) containing 10% heat-inactivated fetal bovine serum (FBS, Bio-Whittaker, Inc.), 1% L-glutamine, 1% sodium pyruvate, 50 U/mL penicillin, and 50 μg/mL streptomycin. The cells were maintained at 37°C in a humidified atmosphere with 5% CO_2_. SKBR3 breast cancer cells were obtained from the American Type Culture Collection (ATCC) and were routinely grown in Improved MEM (IMEM; Biosource International) supplemented with 10% FBS and 1% L-glutamine, 1% sodium pyruvate, 50 U/mL penicillin, and 50 μg/mL streptomycin. Cells were maintained at 37°C in a humidified atmosphere of 95% air and 5% CO_2_. Cells were screened periodically for *Mycoplasma* contamination.

### Lentiviral transduction

Pre-packaged lentiviral particles that either encoded a non-targeting shRNA (negative shRNA, sc-108080) or sequences specifically targeting the human *ATG12* gene were purchased from a commercial provider (Santa Cruz Biotechnology). For viral infection of JIMT1 cells, the regular medium was replaced with culture medium containing 5 μg/mL polybrene (Santa Cruz Biotechnology, sc-124220). JIMT1 cells were then exposed to lentiviruses for 48 h. Because the lentiviral shRNA particles also encode a puromycin resistance gene for transduction selection, the cells were then washed and grown in culture medium containing 10 μg/mL puromycin dihydrochloride (Sigma, P9620) for an additional 72 h. The JIMT1 cells were allowed to recover and proliferate for at least 1 week before any experimental procedures and were then analyzed. To monitor the lentiviral transduction efficiency and transgene expression for the duration of the experiment, we incubated additional subsets of JIMT1 cells with lentiviral particles encoding a green fluorescence protein (GFP) reporter (sc-108084). Transduction efficiency (> 90%) was obtained as the ratio of the number of GFP-positive cells to the total number of cells from five random visual fields from three independent culture experiments.

### Quantitative real-time polymerase chain reaction (qRT-PCR)

Total RNA was extracted from cell cultures using a Qiagen RNeasy kit and Qiash redder columns according to the manufacturer's instructions. One microgram of total RNA was reverse-transcribed into cDNA with a Reaction Ready™ First Strand cDNA Synthesis Kit (SABiosciences) and applied to the Human Autophagy RT² Profiler™ PCR Array (PAHS-084, 96-well format) following the SABiosciences RT-PCR manual. Plates were processed in an Applied Biosystems 7500 Fast Real-Time PCR System Applied Biosystems, using automated baseline and threshold cycle detection. Data were interpreted with SABiosciences’ web-based PCR array analysis tool.

### Metabolic status assessment (MTT-based cell viability assays)

Cell viability was determined using a standard colorimetric MTT (3-4,5-dimethylthiazol-2-yl-2, 5-diphenyl-tetrazolium bromide) reduction assay. Exponentially growing cells were harvested by trypsinization, seeded at a concentration of ~2.5 × 10^3^ cells/200 μL/well into 96-well plates, and allowed to attach overnight. The medium was then removed, and fresh medium along with various concentrations of HER1/2 targeting drugs were added to the cultures as specified. Control cells without drugs were cultured in parallel using the same conditions with comparable media changes. Drugs were not renewed during the entire period of cell exposure. Following treatment (5 days), the medium was removed and replaced with fresh drug-free medium (100 μL/well), and MTT (5 mg/mL in PBS) was added to each well at a 1/10 volume. After incubation for 2–3 h at 37°C, the supernatants were carefully aspirated, 100 μL of DMSO were added to each well, and the plates were agitated to dissolve the crystal product. The optical density (OD) was measured at 570 nm in a multi-well plate reader (Model Anthos Labtec 2010 1.7 reader). The cell viability effects resulting from the exposure of cells to HER1/2-targeting drugs were analyzed as percentages of the control cell absorbances, which were obtained from control wells treated with appropriate concentrations of the agents’ vehicles, which were processed simultaneously. For each treatment, cell viability was evaluated as a percentage using the following equation: (OD_570_ of treated sample/OD_570_ of untreated sample) × 100. Breast cancer cell sensitivity to HER1/2-targeting drugs was expressed in terms of the concentration of drug required to decrease cell viability by 50% (IC_50_ value). Because the percentage of control absorbance was considered to be the surviving fraction of cells, the IC_50_ value was defined as the concentration of HER1/2-targeting drug that produced a 50% reduction in the control absorbance (by interpolation), respectively. The degree of sensitization to HER1/2-targeting drugs by shRNA-driven silencing of *ATG12* was evaluated by dividing the IC_50_ values of the JIMT1 parental cells by those obtained in control-shRNA/JIMT1 cells or ATG12-shRNA/JIMT1 cells.

### Xenograft studies

To produce xenografts, approximately 5 × 10^6^ JIMT1 parental cells, control-shRNA/JIMT1 cells or ATG12-shRNA/JIMT1 cells were injected subcutaneously into the dorsal flanks of female athymic nude mice (four to five weeks old, 23 to 25 g; Harlan Laboratories, France). The animals were randomized into two groups, with five animals in each group: control (vehicle) and trastuzumab-treated. Trastuzumab (5 mg/kg) was administered intraperitoneally (i.p.) once per week. The mice were weighed once per week after dosing, the tumor sizes were measured daily with electronic calipers, and the tumor volumes were calculated with the following formula: volume (mm^3^) = length × width^2^ × 0.5. The experiments were approved by the Institutional Animal Care and Use Committee (IACUC) of the Institut d'Investigació Biomèdica de Bellvitge (IDIBELL; Animal Use Protocol #6302 authorized by the Animal Experimental Commission of the Catalan Government, Barcelona, Spain).
